# Preeclampsia, Hypoxia, Thrombosis, and Inflammation

**DOI:** 10.1155/2012/374047

**Published:** 2011-12-11

**Authors:** Amir A. Shamshirsaz, Michael Paidas, Graciela Krikun

**Affiliations:** Department of Obstetrics, Gynecology and Reproductive Sciences, School of Medicine, Yale University, New Haven, CT 06520, USA

## Abstract

Reductions in uteroplacental flow initiate a cascade of molecular effects leading to hypoxia, thrombosis, inflammation, and endothelial cell dysfunction resulting in untoward pregnancy outcomes. In this review, we detail these effects and their relationship to preeclampsia (PE) and intrauterine growth restriction (IUGR).

## 1. Introduction

PE is universally defined as hypertension and significant proteinuria developed at or after 20 weeks of pregnancy in an otherwise normotensive woman [1–3]. PE is a multisystem disorder which complicates 3–14% of all pregnancies and about 5–8% of pregnancies in the United States [1]. The disease is mild in 75% of cases in the United States, and severe in 25% of cases [4]. Ten percent of PE occurs in pregnancies less than 34 weeks of gestation. The incidence of PE has risen in the USA in the last decades [5]. This finding might be related to an increased prevalence of predisposing disorders, such as maternal age, chronic hypertension, diabetes, prepregnancy obesity, and multiple births [5–7]. Overall, 10%–15% of direct maternal deaths are associated with PE in low- and middle-income countries and the proportion is similar in high-income countries [8, 9]. Furthermore, severe PE is a major cause of maternal morbidity (i.e., stroke and liver rupture) and negative long-term outcomes (i.e., cardiovascular disease and diabetes mellitus) as well as adverse perinatal effects, such as prematurity and intrauterine growth restriction [5, 10].

 While much research has been devoted toward this topic, the cause of PE still remains elusive. Two different theories have emerged: (1) vascular-ischemic origin of PE and (2) impaired immune response [11]. A current hypothesis unifies these concepts where an altered immune response leads to disturbed placental function early in pregnancy with consequent syncytiotrophoblast ischemia and shedding of products that extensively damage endothelial integrity. This in turn results in an exponential production of multiple factors such as cytokines and growth factors leading to the clinical manifestations of PE [11]. How the immune response can activate the cascade process is still unknown but it is proposed to act in synergy with additional exacerbating factors such as predisposing maternal and ambient factors [12].

## 2. Angiogenic Factors and PE

Angiogenic factors and their receptors are important regulators of placental vascular development [13]. The most widely studied serum markers for PE, to date, are vascular endothelial growth factor (VEGF) and placental growth factor (PlGF). Antagonists include soluble fms-like tyrosine kinase 1 (sFlt-1, also known as sVEGFR1), and soluble endoglin (sEng) [13].

sFlt-1 is a truncated splice variant of the membrane-bound Flt1; it consists of the extracellular binding domain without the intracellular signaling domain. Several studies demonstrated the association of increased sFlt-1 with PE [14, 15].

Evidence for the involvement of sFlt1 in the occurrence of PE was initially provided by an animal model in which gravid rats were infected with a recombinant adenovirus encoding sFlt1 and compared to animals infected with the empty control adenovirus. The animals infected with sFlt1 developed a syndrome highly reminiscent of human PE: hypertension and proteinuria due to a glomerular endotheliosis [14, 16].

Similarly, sEng is a truncated form of receptor for two subtypes of transforming growth factor beta (TGF*β*) specifically, TGF*β*1 and TGF*β*2 which are highly expressed by vascular endothelial cells and syncytiotrophoblasts. Like sFlt1, soluble endoglin (sEng) is an antiangiogenic factor capable of inhibiting capillary tube formation *in vitro* [17]. Soluble Eng also increases vascular permeability; overexpression of both sFlt1 and sEng in rodents results in capillary permeability in the lungs, kidneys, and liver [18]. Overexpression of both sEng and sFlt1 in pregnant rats develops nephrotic-range proteinuria, severe hypertension, biochemical evidence of HELLP (“H” for hemolysis, “EL” for elevated liver enzymes, and “LP” for low platelet count), and intrauterine growth restriction of the pups [19, 20].

PlGF concentration during pregnancy increases during the first 30 weeks of gestation, and then decreases [13]. Longitudinal studies have shown that a relatively low PlGF concentration (which could be explained by a high sFlt1 concentration) is also a characteristic feature of PE. Decreased levels of urinary PlGF and PIGF : sFlt-1 ratio during mid-gestation have been proposed as a predictive model for development of clinical PE, and quantification of sFlt-1 levels has correlated directly with severity of disease and inversely with time to onset of proteinuria and hypertension [21].

The exact molecular basis for placental dysregulation of these factors remains unknown but hypoxia is likely an important regulator [22]. Other factors such as alterations in the renin-angiotensin-aldosterone axis, immune maladaption, excessive shedding of trophoblast debris, oxidative stress, and genetic factors likely contribute to the pathogenesis of the abnormal placentation [22]. To date the most successful treatment for PE is delivery.

## 3. Preeclapmpsia and Cathecol-O-methyltrasferase

Cathecol-O-methyltrasferase (COMT) catalyzes the O-methylation of various circulating hormones such as catecholamines and catecholestrogens [23, 24]. In the placenta, COMT metabolizes certain forms of circulating estradiols to the molecule 2-methyl estradiol (2-ME). This estradiol metabolite has several effects, one being the destabilization of hypoxia-inducible factor- (HIF-) 1*α*3 in the cytoplasm. HIFs are heterodimeric proteins that mediate the effects of hypoxia on gene expression by upregulating transcription of target genes including sFlt1 [25]. This role of COMT in maintaining oxygen balance suggests that COMT might somehow be involved in the pathogenesis of PE.

To better elucidate the role of COMT in PE, a genetic COMT knockout (COMT−/−) mouse model was recently developed. Interestingly, pregnant COMT−/− mice developed a PE-like phenotype characterized by proteinuria, increased blood pressure, and histopathological changes in the placenta and kidney. This phenotype was accompanied by lower plasma concentrations of 2-ME and higher placental protein levels of HIF-1a [26]. Restoration of 2-ME in COMT−/− mice decreased HIF-1a and ameliorated the preeclamptic phenotype [26]. Together, these results suggest that COMT and 2-ME deficiency might play a significant role in the development of PE.

The exact mechanism by which 2-ME prevents PE remains unknown. Although 2-ME was found to suppress HIF-1a and sFlt1, recent experiments suggest that there might be several other mechanisms through which 2-ME promotes vascular health [27].

Lastly, recent evidence suggests that 2-ME is necessary for cytotrophoblast invasion of the maternal decidua and therefore contributes to the prevention of PE by promoting normal placental vascular formation [28]. Further studies on this topic will be required to ascertain a more exact mechanism of action.

## 4. PE and Complement Factors

 The complement system, composed of over 30 proteins that act in concert to protect the host against invading organisms, initiates inflammation and tissue injury [29] and normally has a protective role toward off-infection. Complement activation promotes chemotaxis of inflammatory cells and generates proteolytic fragments that enhance phagocytosis by neutrophils and monocytes. There is scant information about complement activation in normal and abnormal human pregnancy [30]. During normal gestation, serum levels of C3, C4, and total hemolytic complement (CH50) gradually increase 10%–50% [31]. Studies have shown significant elevations in levels of Bb, C3a, C4d, and soluble C5b-9 in preeclampsia, indicating excess activation of both the classical and alternative complement pathway [32]. Lynch et al. conducted a large prospective study in human pregnancy to investigate whether elevated levels of complement activation fragment Bb (reflecting alternative complement pathway activation) at a single point in early pregnancy (less than 20 weeks gestation) were predictive of preeclampsia later in pregnancy. Adjusted for other risk factors, women with higher levels of Bb in early pregnancy were almost four times more likely to develop preeclampsia later in pregnancy compared with women with levels less than the top decile in early pregnancy [33]. Products of complement pathway have been found in deciduas, chorionic villi, and as subendothelial deposits in vessel walls in normal and preeclampsia [34, 35]. A recent study reported that the presence in C5b-9 MAC on trophoblasts was associated with fibrin deposits at sites of villous injury *in vivo* in normal placentas, but especially in placentas from pregnancies complicated by IUGR or preeclampsia [36]. It has been demonstrated that in normal pregnancies, complement regulatory proteins that are highly expressed on trophoblast membranes prevent excessive complement activation (membrane cofactor protein [MCP (CD46)], decay accelerating factor [DAF (CD55)], and CD59)) as well as circulating complement regulatory proteins (complement factor H [CFH], C4b binding protein, and complement factor I [CFI]) [37, 38]. Defective regulation of the complement system allows for the excessive complement activation that leads to placental damage, abnormal placental development, generalized endothelial activation, and the release of antiangiogenic factors toxic to the fenestrated endothelium of glomeruli, the choroid plexus, and liver sinusoids—a sequence of events that culminates in clinical preeclampsia [39]. The link between complement activation and pathogenic events in preeclampsia identifies potential biomarkers to predict patients at risk for preeclampsia and new targets to prevent its complications.

## 5. PE and Coagulation Factors

As implied in the preceding section, insufficient and/or inadequate trophoblastic invasion of the maternal spiral arteries leads to reduced uteroplacental blood flow causing focal decidual hypoxia, and thus VEGF [40–42]. This in turn results in activation of the decidual endothelial cells and the aberrant expression of tissue factor (TF) [40, 43]. In addition, under pathological conditions, other cells, such as macrophages present in the maternal fetal interface, can also generate TF. TF generates thrombin that further induces endothelial cell TF expression and inflammatory cytokines [40]. Both VEGF and TF induce aberrant angiogenesis and poor vessel maintenance reflected by endothelial cell fenestrations and induction of a prothrombotic surface and uteroplacental vascular insufficiency [44].

Under physiological conditions, TF is not expressed by endothelial cells. By contrast, endothelial TF expression is observed in pathologic conditions such as sepsis, atherosclerosis, and rapid but malformed vessel growth associated with malignancies and allograft rejection [45–47]. Indeed, expression of TF by the endothelium is a pathological consequence of cross-talk between coagulation and inflammatory cytokines [45]. Expression of TF transforms endothelial cell membrane from an anticoagulant to a procoagulant surface and promotes intravascular thrombosis [48, 49]. Interestingly, the uteroplacental vasculature of pregnancies complicated by PE and IUGR display vascular features similar to those seen in allograft rejection [50]. A recent study by Di Paolo et al. demonstrated high expression of TF mRNA in the vascular endothelium of vessels in the decidua basalis of pregnancies complicated by PE and IUGR but not in normal pregnancies [51].

Tissue factor, the critical initiator of the coagulation cascade, is induced during pregnancy in the maternal decidua [52, 53] and is plentiful in the placenta and amniotic fluid [54–57]. It has been well documented that patients with diseases leading to aberrantly increased circulating TF expression have a much higher rate of vascular thromboembolism (VTE) [58–61]. Specifically, patients are at risk for cardiovascular diseases, sepsis, hematologic, and coagulation disorders such as disseminated intravascular coagulation [62–67]. In these diseases, as well as in cancer and diabetes, plasma TF is related to increased blood thrombogenicity [67]. Interestingly, plasma levels of thrombomodulin activity, tissue factor activity, and procoagulant phospholipids were significantly elevated in women with PE versus normal pregnant and nonpregnant women [68]. Moreover, the circulating TF has been identified on circulating microparticles (MPs). These MPs are small vesicles released from injured or activated cells, primarily leukocytes and endothelial cells [58, 69]. TF-bearing MPs in pregnancy also arise from syncytiotrophoblasts [70] and excess syncytiotrophoblast microparticles have been found in the circulation of women with PE [71]. Unfortunately, VTE is one of the leading causes of morbidity and mortality during pregnancy [72]. A higher prevalence of risk factors for VTE has been found in women with PE and fetal loss [73].The principal factors that result in venous thrombosis consist of the classic triad of Virchow: venous stasis, vascular damage, and hypercoagulability, all of which occur during normal pregnancies [74]. A shift of the hemostatic balance in the direction of hypercoagulability during pregnancy is believed to be evolutionarily advantageous in reducing hemorrhage, however, this could also contribute to the increased risk of venous thromboembolic processes [75]. Studies from our laboratory have demonstrated that TF is normally expressed by decidual cells [76]. Indeed, immunohistochemical staining for TF has been observed in the decidualized endometrial stromal cells, but not in endothelial cells [77–79]. In contrast, we noted that basal plate uteroplacental vessel segments from cases of low birth weight with villous evidence of maternal uteroplacental malperfusion had increased percent of strong positive endothelial immunostaining for TF [40].

## 6. Thrombin Activation of Endometrial Endothelial Cells

Vascular injury initiates clotting when plasma-derived factor VII binds to the extracellular domain of perivascular cell membrane-bound TF. The resulting TF/VIIa complex promotes hemostasis via a series of changes initiated by the extracellular cleavage of prothrombin to thrombin [76]. The aberrant expression of TF in vessels from deciduas with activated endothelium led us to study this phenomenon in further detail. We proposed that aberrantly expressed TF in decidual vessels would in turn result in thrombin production and further endothelial activation. *In vitro* experiments were carried out with cultured human endometrial endothelial cells (HEECs) [40]. Treatment with 2.5 U of thrombin demonstrated the induction of TF protein and mRNA expression [40]. Moreover, thrombin-induced inflammatory cytokine expression in HEECs. Specifically thrombin significantly upregulated mRNA expression for regulated upon activation, normal T-cell expressed, and secreted (RANTES) chemokine, growth-related oncogene (Gro)-*β*, Gro-*γ*, granulocyte chemotactic protein (GCP)-2, granulocyte-macrophage colony stimulating factor (GM-CSF), monocyte chemotactic protein-1 (MCP-1), interleukin-8 (IL-8), and macrophage inflammatory protein 3*α* (MIP3*α*) [40]. This increase in proinflammatory chemokines are expected to result in endothelial dysfunction or inappropriate endothelial cell activation which are the most common clinical manifestations in PE, including enhanced endothelial-cell permeability and platelet aggregation [80]. Moreover, the overexpression of these chemokines is consistent with observations made in our laboratory [81, 82] as well as others [83, 84] indicating that the preeclamptic decidua contains an excess of macrophages [82].

## 7. Summary

To date, no accurate test exists for predicting PE. In recent years, it has become accepted that early-onset and late-onset PEs are associated with different biochemical, histological, and clinical features [85]. Moreover, markers such as sFLT, sEng, products of fetal and placental origin, markers of renal or endothelial damage, or markers of oxidative stress are secondary to the pathophysiological changes that precede the clinical onset of PE [85]. Nonetheless, a combination of markers may increase the detection accuracy earlier in the pregnancy and hopefully allow for more effective prophylactic strategies.
